# 3-(3-Bromo­phenyl­sulfin­yl)-5-cyclo­hexyl-2-methyl-1-benzo­furan

**DOI:** 10.1107/S160053681400316X

**Published:** 2014-02-15

**Authors:** Hong Dae Choi, Pil Ja Seo, Uk Lee

**Affiliations:** a Department of Chemistry, Dongeui University, San 24 Kaya-dong, Busanjin-gu, Busan 614-714, Republic of Korea; b Department of Chemistry, Pukyong National University, 599-1 Daeyeon 3-dong, Nam-gu, Busan 608-737, Republic of Korea

## Abstract

In the title compound, C_21_H_21_BrO_2_S, the cyclo­hexyl ring adopts a chair conformation. The dihedral angle between the mean plane [r.m.s. deviation = 0.178 (2) Å] of the benzo­furan ring system and the mean plane of the 3-bromo­phenyl ring is 86.52 (6)°. In the crystal, mol­ecules are linked by weak C—H⋯O and C—H⋯π hydrogen bonds, and by a slipped π–π inter­action between the furan rings of neighbouring mol­ecules [centroid–centroid distance = 3.518 (3) Å, inter­planar distance = 3.471 (3) Å and slippage = 0.573 (3) Å], resulting in a three-dimensional network.

## Related literature   

For background information and the crystal structures of related compounds, see: Choi *et al.* (2011 ▶, 2012*a* ▶,*b* ▶).
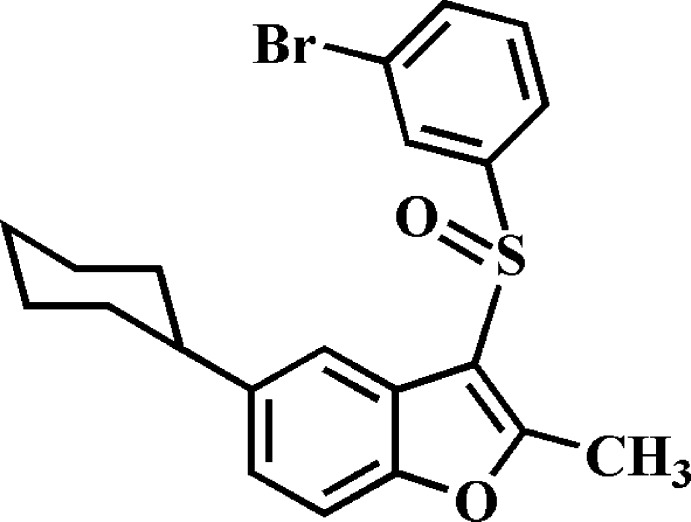


## Experimental   

### 

#### Crystal data  

C_21_H_21_BrO_2_S
*M* *_r_* = 417.35
Monoclinic, 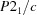

*a* = 17.6432 (9) Å
*b* = 8.9425 (5) Å
*c* = 12.4776 (7) Å
β = 108.277 (3)°
*V* = 1869.33 (18) Å^3^
*Z* = 4
Mo *K*α radiation
μ = 2.32 mm^−1^
*T* = 173 K
0.33 × 0.25 × 0.18 mm

#### Data collection  

Bruker SMART APEXII CCD diffractometer
Absorption correction: multi-scan (*SADABS*; Bruker, 2009 ▶) *T*_min_ = 0.482, *T*_max_ = 0.746
13952 measured reflections
3290 independent reflections
2635 reflections with *I* > 2σ(*I*)
*R*_int_ = 0.035

#### Refinement  

*R*[*F* ^2^ > 2σ(*F* ^2^)] = 0.031
*wR*(*F* ^2^) = 0.081
*S* = 1.02
3290 reflections
227 parameters
H-atom parameters constrained
Δρ_max_ = 0.33 e Å^−3^
Δρ_min_ = −0.35 e Å^−3^

### 

Data collection: *APEX2* (Bruker, 2009 ▶); cell refinement: *SAINT* (Bruker, 2009 ▶); data reduction: *SAINT*; program(s) used to solve structure: *SHELXS97* (Sheldrick, 2008 ▶); program(s) used to refine structure: *SHELXL97* (Sheldrick, 2008 ▶); molecular graphics: *ORTEP-3 for Windows* (Farrugia, 2012 ▶) and *DIAMOND* (Brandenburg, 1998 ▶); software used to prepare material for publication: *SHELXL97*.

## Supplementary Material

Crystal structure: contains datablock(s) I. DOI: 10.1107/S160053681400316X/gk2602sup1.cif

Structure factors: contains datablock(s) I. DOI: 10.1107/S160053681400316X/gk2602Isup2.hkl

Supporting information file. DOI: 10.1107/S160053681400316X/gk2602Isup3.cml

CCDC reference: 986408

Additional supporting information:  crystallographic information; 3D view; checkCIF report

## Figures and Tables

**Table 1:** Hydrogen-bond geometry (Å, °) *Cg*1 is the centroid of the C2–C7 benzene ring.

*D*—H⋯*A*	*D*—H	H⋯*A*	*D*⋯*A*	*D*—H⋯*A*
C15—H15*A*⋯O1^i^	0.98	2.58	3.358 (3)	136
C21—H21⋯O2^ii^	0.95	2.45	3.306 (3)	151
C15—H15*C*⋯*Cg*1^iii^	0.98	2.82	3.502 (3)	127

Symmetry codes: (i) 
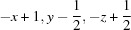
; (ii) 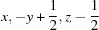; (iii) 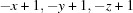.

**Fig. 1.**
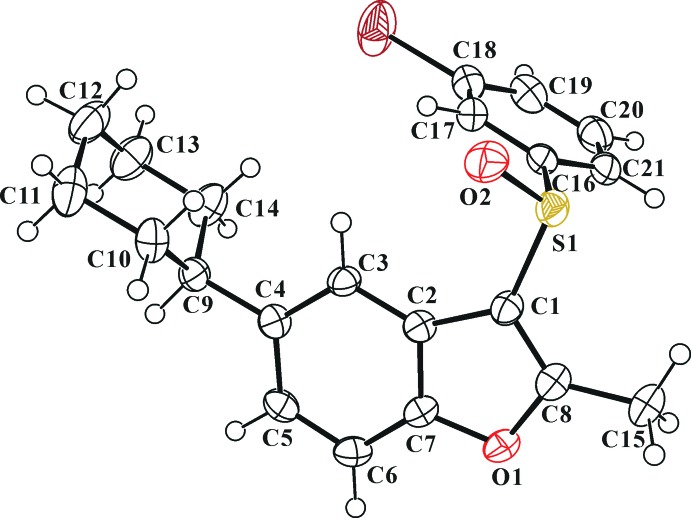
The molecular structure of the title compound with the atom numbering scheme. Displacement ellipsoids are drawn at the 50% probability level. H atoms are presented as small spheres of arbitrary radius.

**Fig. 2.**
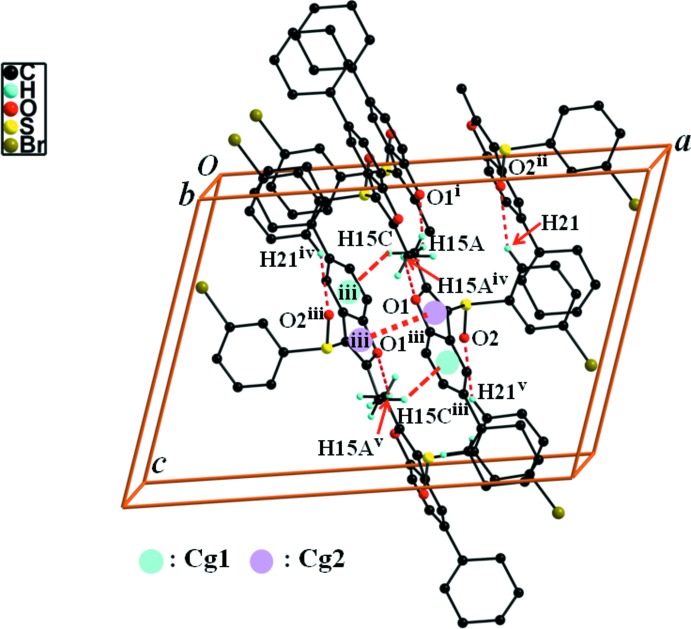
A view of the C—H···O, C—H···π and π···π interactions (dotted lines) in the crystal structure of the title compound. H atoms non-participating in hydrogen bonding were omitted for clarity. [Symmetry codes : (i) - x + 1, y - 1/2, - z + 1/2 ; (ii) x, - y + 1/2, z - 1/2; (iii) - x + 1, - y + 1, - z + 1; (iv) - x + 1, y + 1/2, - z + 1/2; (v) x, - y + 1/2, z + 1/2.]

## supplementary crystallographic information

### 1. Comment

As a part of our continuing study of 5-cyclohexyl-2-methyl-1-benzofuran derivatives containing 4-fluorophenylsulfinyl (Choi *et al.*, 2011), 4-bromophenylsulfinyl (Choi *et al.*, 2012*a*) and 4-methylphenylsulfinyl (Choi *et al.*, 2012*b*) substituents in 3-position, we report herein the crystal structure of the title compound.

In the title molecule (Fig. 1), the cyclohexyl ring has a chair conformation. The benzofuran ring system is essentially planar, with a mean deviation of 0.178 (2) Å from the least-squares plane defined by the nine constituent atoms. The 3-bromophenyl ring is essentially planar, with a mean deviation of 0.005 (2) Å from the least-squares plane defined by the six constituent atoms. The dihedral angle formed by the benzofuran ring system and the 3-bromophenyl ring is 86.52 (6)°. In the crystal structure (Fig. 2), molecules are connected by C—H···O and C—H···π hydrogen bonds (Table 1, Cg1 is the centroid of the C2–C7 benzene ring). The crystal packing (Fig. 2) also exhibits a slipped π···π interaction between the furan rings of neighbouring molecules, with a Cg2···Cg2^iii^ distance of 3.518 (3) Å and an interplanar distance of 3.471 (3) Å resulting in a slippage of 0.573 (3) Å (Cg2 is the centroid of the C1/C2/C7/O1/C8 furan ring).

### 2. Experimental

3-Chloroperoxybenzoic acid (77%, 224 mg, 1.0 mmol) was added in small portions to a stirred solution of 3-(3-bromophenylsulfanyl)-5-cyclohexyl-2-methyl-1-benzofuran(361 mg, 0.9 mmol) in dichloromethane (40 mL) at 273 K. After being stirred at room temperature for 4h, the mixture was washed with saturated sodium bicarbonate solution and the organic layer was separated, dried over magnesium sulfate, filtered and concentrated at reduced pressure. The residue was purified by column chromatography (hexane–ethyl acetate, 2:1 v/v) to afford the title compound as a colorless solid [yield 71%, m.p. 415–416 K; *R*_f_ = 0.47 (hexane–ethyl acetate, 2:1 v/v)]. Single crystals suitable for X-ray diffraction were prepared by slow evaporation of a solution of the title compound in ethyl acetate at room temperature.

### 3. Refinement

All H atoms were positioned geometrically and refined using a riding model, with C—H = 0.95 Å for aryl, 1.00 Å for methine, 0.99 Å for methylene and 0.98 Å for methyl H atoms, respectively. *U*_iso_ (H) = 1.2*U*_eq_ (C) for aryl, methine and methylene, and 1.5*U*_eq_ (C) for methyl H atoms. The positions of methyl hydrogens were optimized rotationally.

### Crystal data

**Table:**

C_21_H_21_BrO_2_S	*F*(000) = 856
*M**_r_* = 417.35	*D*_x_ = 1.483 Mg m^−^^3^
Monoclinic, *P*2_1_/*c*	Melting point = 415–416 K
Hall symbol: -P 2ybc	Mo *K*α radiation, λ = 0.71073 Å
*a* = 17.6432 (9) Å	Cell parameters from 5628 reflections
*b* = 8.9425 (5) Å	θ = 2.4–27.9°
*c* = 12.4776 (7) Å	µ = 2.32 mm^−^^1^
β = 108.277 (3)°	*T* = 173 K
*V* = 1869.33 (18) Å^3^	Block, colourless
*Z* = 4	0.33 × 0.25 × 0.18 mm

### Data collection

**Table:**

Bruker SMART APEXII CCD diffractometer	3290 independent reflections
Radiation source: rotating anode	2635 reflections with *I* > 2σ(*I*)
Graphite multilayer monochromator	*R*_int_ = 0.035
Detector resolution: 10.0 pixels mm^-1^	θ_max_ = 25.0°, θ_min_ = 2.4°
φ and ω scans	*h* = −20→20
Absorption correction: multi-scan (*SADABS*; Bruker, 2009)	*k* = −9→10
*T*_min_ = 0.482, *T*_max_ = 0.746	*l* = −14→14
13952 measured reflections	

### Refinement

**Table:**

Refinement on *F*^2^	Primary atom site location: structure-invariant direct methods
Least-squares matrix: full	Secondary atom site location: difference Fourier map
*R*[*F*^2^ > 2σ(*F*^2^)] = 0.031	Hydrogen site location: difference Fourier map
*wR*(*F*^2^) = 0.081	H-atom parameters constrained
*S* = 1.02	*w* = 1/[σ^2^(*F*_o_^2^) + (0.0334*P*)^2^ + 1.1209*P*] where *P* = (*F*_o_^2^ + 2*F*_c_^2^)/3
3290 reflections	(Δ/σ)_max_ = 0.001
227 parameters	Δρ_max_ = 0.33 e Å^−^^3^
0 restraints	Δρ_min_ = −0.35 e Å^−^^3^

### Special details

**Table:**

Geometry. All esds (except the esd in the dihedral angle between two l.s. planes) are estimated using the full covariance matrix. The cell esds are taken into account individually in the estimation of esds in distances, angles and torsion angles; correlations between esds in cell parameters are only used when they are defined by crystal symmetry. An approximate (isotropic) treatment of cell esds is used for estimating esds involving l.s. planes.
Refinement. Refinement of F^2^ against ALL reflections. The weighted R-factor wR and goodness of fit S are based on F^2^, conventional R-factors R are based on F, with F set to zero for negative F^2^. The threshold expression of F^2^ > 2sigma(F^2^) is used only for calculating R-factors(gt) etc. and is not relevant to the choice of reflections for refinement. R-factors based on F^2^ are statistically about twice as large as those based on F, and R- factors based on ALL data will be even larger.

### Fractional atomic coordinates and isotropic or equivalent isotropic displacement parameters (Å^2^)

**Table:**

	*x*	*y*	*z*	*U*_iso_*/*U*_eq_	
Br1	0.957798 (17)	0.38271 (5)	0.67946 (3)	0.07366 (16)	
S1	0.63694 (3)	0.26562 (6)	0.46130 (5)	0.03447 (16)	
O1	0.53393 (8)	0.66112 (17)	0.40386 (12)	0.0319 (4)	
O2	0.64909 (10)	0.19977 (18)	0.57522 (14)	0.0427 (4)	
C1	0.60397 (12)	0.4510 (2)	0.46411 (18)	0.0282 (5)	
C2	0.63088 (12)	0.5613 (2)	0.55301 (17)	0.0256 (5)	
C3	0.68650 (12)	0.5662 (2)	0.66079 (18)	0.0273 (5)	
H3	0.7173	0.4802	0.6915	0.033*	
C4	0.69659 (12)	0.6982 (2)	0.72299 (18)	0.0280 (5)	
C5	0.64951 (13)	0.8227 (3)	0.67603 (19)	0.0319 (5)	
H5	0.6567	0.9126	0.7186	0.038*	
C6	0.59308 (13)	0.8197 (3)	0.57033 (19)	0.0336 (5)	
H6	0.5612	0.9046	0.5399	0.040*	
C7	0.58539 (12)	0.6878 (2)	0.51138 (18)	0.0274 (5)	
C8	0.54640 (12)	0.5152 (3)	0.37802 (18)	0.0296 (5)	
C9	0.75683 (13)	0.7056 (3)	0.84023 (18)	0.0316 (5)	
H9	0.7543	0.8085	0.8704	0.038*	
C10	0.73622 (14)	0.5948 (3)	0.92025 (19)	0.0380 (6)	
H10A	0.6824	0.6180	0.9245	0.046*	
H10B	0.7348	0.4924	0.8895	0.046*	
C11	0.79601 (17)	0.5999 (3)	1.0382 (2)	0.0499 (7)	
H11A	0.7937	0.6990	1.0723	0.060*	
H11B	0.7820	0.5231	1.0860	0.060*	
C12	0.87977 (16)	0.5719 (3)	1.0348 (2)	0.0515 (7)	
H12A	0.8834	0.4687	1.0081	0.062*	
H12B	0.9179	0.5810	1.1120	0.062*	
C13	0.90249 (16)	0.6819 (4)	0.9573 (2)	0.0512 (7)	
H13A	0.9560	0.6560	0.9528	0.061*	
H13B	0.9054	0.7839	0.9892	0.061*	
C14	0.84220 (14)	0.6799 (3)	0.8393 (2)	0.0433 (6)	
H14A	0.8451	0.5822	0.8035	0.052*	
H14B	0.8564	0.7586	0.7931	0.052*	
C15	0.49706 (14)	0.4623 (3)	0.26513 (19)	0.0373 (6)	
H15A	0.5103	0.3578	0.2551	0.056*	
H15B	0.5078	0.5241	0.2067	0.056*	
H15C	0.4404	0.4699	0.2589	0.056*	
C16	0.73449 (14)	0.3121 (2)	0.45342 (18)	0.0319 (5)	
C17	0.79756 (14)	0.3239 (3)	0.55204 (19)	0.0353 (5)	
H17	0.7903	0.3067	0.6233	0.042*	
C18	0.87176 (15)	0.3615 (3)	0.5440 (2)	0.0418 (6)	
C19	0.88362 (16)	0.3840 (3)	0.4411 (2)	0.0451 (6)	
H19	0.9350	0.4092	0.4373	0.054*	
C20	0.81998 (17)	0.3694 (3)	0.3439 (2)	0.0446 (7)	
H20	0.8278	0.3846	0.2727	0.054*	
C21	0.74495 (16)	0.3330 (3)	0.34816 (19)	0.0378 (6)	
H21	0.7013	0.3224	0.2808	0.045*	

### Atomic displacement parameters (Å^2^)

**Table:**

	*U*^11^	*U*^22^	*U*^33^	*U*^12^	*U*^13^	*U*^23^
Br1	0.03672 (18)	0.1296 (4)	0.0485 (2)	0.00172 (17)	0.00453 (14)	−0.02246 (18)
S1	0.0418 (3)	0.0248 (3)	0.0302 (3)	−0.0027 (2)	0.0018 (3)	−0.0026 (2)
O1	0.0302 (8)	0.0323 (10)	0.0295 (8)	0.0027 (7)	0.0040 (7)	0.0072 (7)
O2	0.0528 (10)	0.0314 (10)	0.0403 (10)	0.0006 (8)	0.0097 (8)	0.0081 (8)
C1	0.0291 (11)	0.0263 (13)	0.0268 (11)	−0.0029 (9)	0.0054 (9)	0.0020 (10)
C2	0.0262 (11)	0.0219 (12)	0.0287 (12)	−0.0036 (9)	0.0085 (9)	0.0008 (9)
C3	0.0277 (11)	0.0225 (12)	0.0288 (12)	0.0012 (9)	0.0046 (9)	0.0028 (9)
C4	0.0283 (11)	0.0252 (13)	0.0309 (12)	−0.0043 (9)	0.0099 (10)	−0.0016 (10)
C5	0.0370 (12)	0.0217 (12)	0.0380 (13)	0.0002 (10)	0.0132 (11)	−0.0040 (10)
C6	0.0335 (12)	0.0271 (13)	0.0392 (13)	0.0071 (10)	0.0100 (11)	0.0060 (11)
C7	0.0249 (11)	0.0282 (13)	0.0279 (11)	0.0000 (9)	0.0065 (9)	0.0047 (10)
C8	0.0288 (11)	0.0300 (13)	0.0289 (12)	−0.0053 (10)	0.0074 (10)	0.0030 (10)
C9	0.0367 (12)	0.0239 (13)	0.0297 (12)	−0.0019 (10)	0.0039 (10)	−0.0051 (10)
C10	0.0412 (14)	0.0461 (16)	0.0284 (12)	−0.0063 (11)	0.0133 (11)	−0.0051 (11)
C11	0.0648 (18)	0.0553 (19)	0.0289 (13)	−0.0076 (14)	0.0135 (13)	−0.0022 (12)
C12	0.0511 (16)	0.0556 (18)	0.0351 (14)	−0.0001 (13)	−0.0049 (13)	0.0036 (13)
C13	0.0372 (14)	0.0610 (19)	0.0454 (16)	−0.0093 (13)	−0.0016 (12)	0.0001 (14)
C14	0.0335 (13)	0.0559 (17)	0.0375 (14)	−0.0107 (12)	0.0067 (11)	0.0049 (12)
C15	0.0336 (12)	0.0450 (16)	0.0275 (12)	−0.0061 (11)	0.0012 (10)	0.0021 (11)
C16	0.0426 (13)	0.0197 (12)	0.0293 (12)	0.0048 (10)	0.0054 (10)	−0.0030 (9)
C17	0.0420 (14)	0.0350 (14)	0.0266 (12)	0.0079 (11)	0.0073 (11)	−0.0029 (10)
C18	0.0408 (14)	0.0444 (16)	0.0372 (14)	0.0054 (11)	0.0078 (11)	−0.0099 (12)
C19	0.0492 (15)	0.0421 (16)	0.0476 (16)	0.0020 (12)	0.0205 (13)	−0.0061 (12)
C20	0.0670 (18)	0.0348 (15)	0.0366 (14)	0.0071 (13)	0.0226 (14)	0.0012 (11)
C21	0.0556 (16)	0.0256 (13)	0.0267 (12)	0.0084 (11)	0.0050 (11)	−0.0009 (10)

### Geometric parameters (Å, º)

**Table:**

Br1—C18	1.894 (2)	C11—C12	1.512 (4)
S1—O2	1.4907 (17)	C11—H11A	0.9900
S1—C1	1.761 (2)	C11—H11B	0.9900
S1—C16	1.803 (2)	C12—C13	1.519 (4)
O1—C8	1.378 (3)	C12—H12A	0.9900
O1—C7	1.385 (3)	C12—H12B	0.9900
C1—C8	1.353 (3)	C13—C14	1.522 (3)
C1—C2	1.449 (3)	C13—H13A	0.9900
C2—C7	1.390 (3)	C13—H13B	0.9900
C2—C3	1.395 (3)	C14—H14A	0.9900
C3—C4	1.393 (3)	C14—H14B	0.9900
C3—H3	0.9500	C15—H15A	0.9800
C4—C5	1.402 (3)	C15—H15B	0.9800
C4—C9	1.516 (3)	C15—H15C	0.9800
C5—C6	1.381 (3)	C16—C17	1.380 (3)
C5—H5	0.9500	C16—C21	1.395 (3)
C6—C7	1.374 (3)	C17—C18	1.386 (4)
C6—H6	0.9500	C17—H17	0.9500
C8—C15	1.482 (3)	C18—C19	1.379 (4)
C9—C14	1.527 (3)	C19—C20	1.376 (4)
C9—C10	1.529 (3)	C19—H19	0.9500
C9—H9	1.0000	C20—C21	1.380 (4)
C10—C11	1.519 (3)	C20—H20	0.9500
C10—H10A	0.9900	C21—H21	0.9500
C10—H10B	0.9900		
			
O2—S1—C1	107.61 (10)	C10—C11—H11B	109.5
O2—S1—C16	106.78 (10)	H11A—C11—H11B	108.1
C1—S1—C16	96.38 (10)	C11—C12—C13	111.5 (2)
C8—O1—C7	106.61 (16)	C11—C12—H12A	109.3
C8—C1—C2	107.62 (19)	C13—C12—H12A	109.3
C8—C1—S1	123.66 (17)	C11—C12—H12B	109.3
C2—C1—S1	128.71 (16)	C13—C12—H12B	109.3
C7—C2—C3	118.9 (2)	H12A—C12—H12B	108.0
C7—C2—C1	104.83 (18)	C12—C13—C14	111.4 (2)
C3—C2—C1	136.2 (2)	C12—C13—H13A	109.3
C4—C3—C2	119.5 (2)	C14—C13—H13A	109.3
C4—C3—H3	120.3	C12—C13—H13B	109.3
C2—C3—H3	120.3	C14—C13—H13B	109.3
C3—C4—C5	119.0 (2)	H13A—C13—H13B	108.0
C3—C4—C9	120.2 (2)	C13—C14—C9	112.5 (2)
C5—C4—C9	120.8 (2)	C13—C14—H14A	109.1
C6—C5—C4	122.6 (2)	C9—C14—H14A	109.1
C6—C5—H5	118.7	C13—C14—H14B	109.1
C4—C5—H5	118.7	C9—C14—H14B	109.1
C7—C6—C5	116.6 (2)	H14A—C14—H14B	107.8
C7—C6—H6	121.7	C8—C15—H15A	109.5
C5—C6—H6	121.7	C8—C15—H15B	109.5
C6—C7—O1	126.27 (19)	H15A—C15—H15B	109.5
C6—C7—C2	123.4 (2)	C8—C15—H15C	109.5
O1—C7—C2	110.36 (19)	H15A—C15—H15C	109.5
C1—C8—O1	110.57 (19)	H15B—C15—H15C	109.5
C1—C8—C15	133.6 (2)	C17—C16—C21	121.4 (2)
O1—C8—C15	115.83 (19)	C17—C16—S1	119.04 (18)
C4—C9—C14	112.37 (19)	C21—C16—S1	119.52 (18)
C4—C9—C10	111.31 (18)	C16—C17—C18	118.0 (2)
C14—C9—C10	110.16 (19)	C16—C17—H17	121.0
C4—C9—H9	107.6	C18—C17—H17	121.0
C14—C9—H9	107.6	C19—C18—C17	121.7 (2)
C10—C9—H9	107.6	C19—C18—Br1	120.2 (2)
C11—C10—C9	112.2 (2)	C17—C18—Br1	118.06 (19)
C11—C10—H10A	109.2	C20—C19—C18	119.1 (2)
C9—C10—H10A	109.2	C20—C19—H19	120.4
C11—C10—H10B	109.2	C18—C19—H19	120.4
C9—C10—H10B	109.2	C19—C20—C21	121.0 (2)
H10A—C10—H10B	107.9	C19—C20—H20	119.5
C12—C11—C10	110.8 (2)	C21—C20—H20	119.5
C12—C11—H11A	109.5	C20—C21—C16	118.7 (2)
C10—C11—H11A	109.5	C20—C21—H21	120.7
C12—C11—H11B	109.5	C16—C21—H21	120.7
			
O2—S1—C1—C8	−139.65 (19)	C7—O1—C8—C15	−179.84 (18)
C16—S1—C1—C8	110.4 (2)	C3—C4—C9—C14	62.5 (3)
O2—S1—C1—C2	40.6 (2)	C5—C4—C9—C14	−118.4 (2)
C16—S1—C1—C2	−69.3 (2)	C3—C4—C9—C10	−61.6 (3)
C8—C1—C2—C7	0.1 (2)	C5—C4—C9—C10	117.5 (2)
S1—C1—C2—C7	179.85 (17)	C4—C9—C10—C11	179.8 (2)
C8—C1—C2—C3	179.3 (2)	C14—C9—C10—C11	54.5 (3)
S1—C1—C2—C3	−0.9 (4)	C9—C10—C11—C12	−56.3 (3)
C7—C2—C3—C4	−1.6 (3)	C10—C11—C12—C13	56.0 (3)
C1—C2—C3—C4	179.3 (2)	C11—C12—C13—C14	−55.0 (3)
C2—C3—C4—C5	0.9 (3)	C12—C13—C14—C9	54.1 (3)
C2—C3—C4—C9	−179.93 (19)	C4—C9—C14—C13	−178.0 (2)
C3—C4—C5—C6	0.2 (3)	C10—C9—C14—C13	−53.3 (3)
C9—C4—C5—C6	−178.9 (2)	O2—S1—C16—C17	−18.3 (2)
C4—C5—C6—C7	−0.7 (3)	C1—S1—C16—C17	92.32 (19)
C5—C6—C7—O1	−178.7 (2)	O2—S1—C16—C21	160.74 (18)
C5—C6—C7—C2	0.0 (3)	C1—S1—C16—C21	−88.7 (2)
C8—O1—C7—C6	179.7 (2)	C21—C16—C17—C18	1.8 (3)
C8—O1—C7—C2	0.8 (2)	S1—C16—C17—C18	−179.20 (18)
C3—C2—C7—C6	1.1 (3)	C16—C17—C18—C19	−1.3 (4)
C1—C2—C7—C6	−179.5 (2)	C16—C17—C18—Br1	178.45 (18)
C3—C2—C7—O1	−179.96 (18)	C17—C18—C19—C20	0.3 (4)
C1—C2—C7—O1	−0.5 (2)	Br1—C18—C19—C20	−179.37 (19)
C2—C1—C8—O1	0.5 (2)	C18—C19—C20—C21	0.1 (4)
S1—C1—C8—O1	−179.36 (14)	C19—C20—C21—C16	0.4 (4)
C2—C1—C8—C15	179.3 (2)	C17—C16—C21—C20	−1.4 (3)
S1—C1—C8—C15	−0.5 (4)	S1—C16—C21—C20	179.61 (18)
C7—O1—C8—C1	−0.8 (2)		

### Hydrogen-bond geometry (Å, º)

Cg1 is the centroid of the C2–C7 benzene ring.

**Table:**

*D*—H···*A*	*D*—H	H···*A*	*D*···*A*	*D*—H···*A*
C15—H15*A*···O1^i^	0.98	2.58	3.358 (3)	136
C21—H21···O2^ii^	0.95	2.45	3.306 (3)	151
C15—H15*C*···*Cg*1^iii^	0.98	2.82	3.502 (3)	127

Symmetry codes: (i) −*x*+1, *y*−1/2, −*z*+1/2; (ii) *x*, −*y*+1/2, *z*−1/2; (iii) −*x*+1, −*y*+1, −*z*+1.
